# Multi-scale movement syndromes for comparative analyses of animal movement patterns

**DOI:** 10.1186/s40462-022-00365-y

**Published:** 2023-10-04

**Authors:** Roland Kays, Ben Hirsch, Damien Caillaud, Rafael Mares, Shauhin Alavi, Rasmus Worsøe Havmøller, Margaret Crofoot

**Affiliations:** 1 Smithsonian Tropical Research Institute, Balboa, Republic of Panama; 2 North Carolina Museum of Natural Sciences, Raleigh, NC USA; 3 Department of Forestry and Environmental Resources, North Carolina State University, Raleigh, NC USA; 4 College of Science and Engineering, James Cook University, Townsville, QLD Australia; 5 Department of Anthropology, University of California, Davis, CA USA; 6 School of Biological Sciences, University of Bristol, Bristol, UK; 7 Department for the Ecology of Animal Societies, Max Planck Institute of Animal Behavior, Constance, Germany; 8 Research and Collections, Natural History Museum of Denmark, University of Copenhagen, Copenhagen, Denmark; 9 Department of Biology, University of Konstanz, Constance, Germany; 10 Center for the Advanced Study of Collective Behavior, University of Konstanz, Constance, Germany

## Abstract

**Background:**

Animal movement is a behavioral trait shaped by the need to find food and suitable habitat, avoid predators, and reproduce. Using high-resolution tracking data, it is possible to describe movement in greater detail than ever before, which has led to many discoveries about the behavioral strategies of particular species. Recently, enough data been become available to enable a comparative approach, which has the potential to uncover general causes and consequences of variation in movement patterns, but which must be scale specific.

**Methods:**

Here we introduce a new multi-scale movement syndrome (MSMS) framework for describing and comparing animal movements and use it to explore the behavior of four sympatric mammals. MSMS incorporates four hierarchical scales of animal movement: (1) fine-scale movement steps which accumulate into (2) daily paths which then, over weeks or months, form a (3) life-history phase. Finally, (4) the lifetime track of an individual consists of multiple life-history phases connected by dispersal or migration events. We suggest a series of metrics to describe patterns of movement at each of these scales and use the first three scales of this framework to compare the movement of 46 animals from four frugivorous mammal species.

**Results:**

While subtle differences exist between the four species in their step-level movements, they cluster into three distinct movement syndromes in both path- and life-history phase level analyses. Differences in feeding ecology were a better predictor of movement patterns than a species’ locomotory or sensory adaptations.

**Conclusions:**

Given the role these species play as seed dispersers, these movement syndromes could have important ecosystem implications by affecting the pattern of seed deposition. This multiscale approach provides a hierarchical framework for comparing animal movement for addressing ecological and evolutionary questions. It parallels scales of analyses for resource selection functions, offering the potential to connect movement process with emergent patterns of space use.

**Supplementary Information:**

The online version contains supplementary material available at 10.1186/s40462-022-00365-y.

## Introduction

**Fig. 1:**
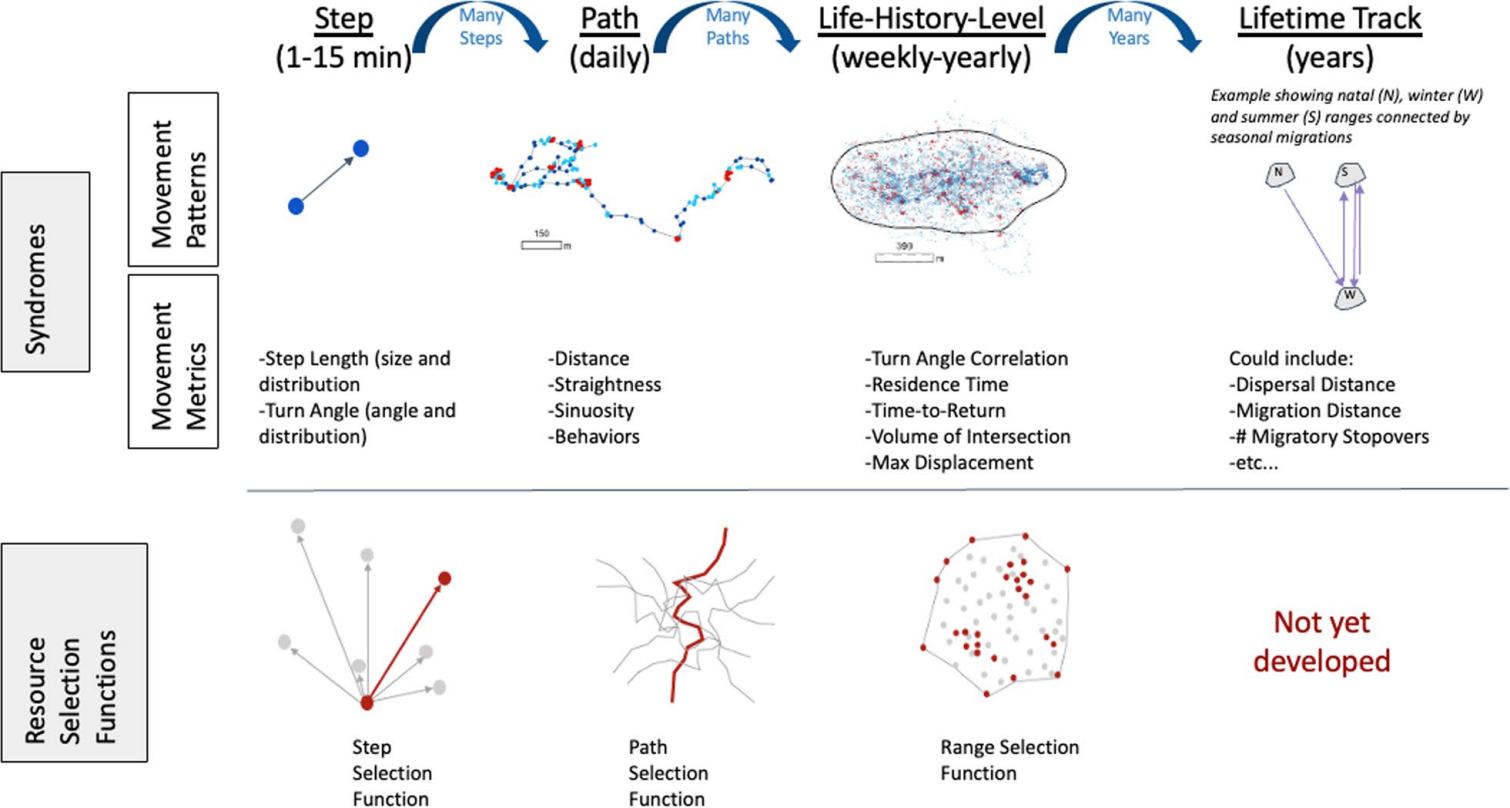
A schematic of the multi-scale movement syndromes (MSMS) framework and how it relates to existing Resource Selection Functions. The syndromes describe the pattern of animal movement while the RSF’s quantify the process as an animal interacts with the surrounding environment (i.e. habitat preferences). The MSMS expands the life-history-level syndromes described by Abrahms et al. [1] to explicitly incorporate the natural hierarchy that emerges as steps form into daily paths, which accumulate into longer-term phases and, eventually, lifetime tracks. This framework allows us to not only describe variation in patterns of animal space-use, but also to investigate at what scale such differences emerge

There is a hierarchical scale of animal movement which is reflected in how animals use their environment (Fig. 1, [3]. At a fine scale, animals move in steps. Limbed animals, of course, take literal steps, but in the analysis of movement data, the term ‘step’ is used to denote the smallest-scale components of a trajectory. This definition of a step can be rooted in the biology of the organism being studied; for example, reflecting the scale at which the animal makes decisions about where to move and the complexity of their movement. More often, however, the scale measured by scientists is somewhat arbitrary and often imposed by the sampling rate of the measurement technology. Steps accumulate into behavioral phases that represent, feeding clusters or traveling, or other activities [37]. These behaviors then accumulate into the path traversed over the course of a day, a natural unit reflected in the circadian rhythms of almost all species [49]. Over weeks or months, paths accumulate into a ‘range’ that we term the life-history phase. If the animal shows a localizing tendency this is known as a home range but some nomadic or dispersing animals might not establish such a stable range [7, 39]. Over an animals lifetime, this scale of movement is made up of multiple life-history phases consisting of ranges and movement between ranges [27]. For example, the life of a typical terrestrial mammal might have three life history movement phases with a natal range followed by a dispersal event that leads to their adult home range. This could be more complicated in migratory animals that also have seasonal home ranges on separate continents separated by migrations, which themselves might have multiple stop-over events [42].

The goal of this paper is to find useful ways to compare the patterns of movement across individuals and species while being cognizant of scale. To that end, we need to think critically about how these scales are chosen and differentiated, how they are affected by the way tracking data is collected, how valid it is to compare them across species, and what biological inferences might be possible at the different scales. There has not been a systematic evaluation of how time interval affects inference of movement steps. While almost certainly system specific and depending on the complexity of the animal’s movement at different time scales, we suggest suggest a lower limit of ~ 1 min and an upper limit of ~ 6 h. Steps of less than 1 min could reflect GPS error rather than actual movement (depending on the technology, [38]), and otherwise would reflect such small-scale movements this analysis would generate more noise than signal. The daily paths generated from steps larger than 6 h would be very imprecise, and only the most coarse-scale behaviors would be distinguishable. Our general suggestion is to select a step interval that is biologically meaningful for the group of species in question, fine enough to resolve behaviors and day ranges, and realistic given technological limitations.

There are a number of approaches to classify consecutive movement steps into behavioral phases which then can then be strung together to form a movement path [15]. However, comparing these phases between species is not straightforward. The behaviors they represent could vary greatly, and the temporal scale of these phases are not consistent. Thus, instead of using these phases as a unit to compare across species, we suggest using measures of behavioral phases as part of the characterization of the daily behavior of an animal, and then compare these daily intervals across species. Daily rhythms are nearly a universal aspect of animal behavior [35], making it a natural temporal scale to compare movement.

Multiple days of movement can be combined into a larger temporal scale we refer to as the life-history phase, which can last for weeks, months, or years. The critical point for comparing these is to make sure individuals are in the same phase. As with behavioral phases, there are empirical approaches to distinguishing range resident species from migrants or dispersers based on movement patterns [17]. Researchers looking to compare multiple life history phases across the lifetimes of animals should consider both range residency, age, and biology of the species to ensure the comparisons are appropriate.

It is useful to consider animal movement at different scales because they reflect different aspects of an animal’s biology, and thus allow us to ask different questions about animal ecology and evolution [37]. Here we suggest that some research questions are better suited to particular scales of movement, and that thinking about movement at different scales helps break apart broad questions into smaller, more testable predictions (Table 1). The sensory perception of a species and its ability to move through the environment will primarily be reflected by step level movement decisions—this is when an individual sees/hears/smells the options available to them and decides where to move based on what it can sense about its environment and the ability to move through it. These factors could also have some effect on daily movement paths if species use long-distance sensing or memory to plan longer distance travel, or if endurance becomes an important limiting factor for daily movement. Movement patterns provide unique opportunities to study animal memory, especially when animals navigate towards known resources [16]. In such cases, memory will affect step level decisions by setting the general direction of small-scale movements in alignment with the direction of longer-term goals [40]. Memory will also shape life-history phases or lifetime tracks when animals remember the locations of seasonal resources or migration routes [25]. A species’ ecological niche and various social factors could be important at all scales—showing how species find food and shelter while avoiding predators and negotiating within and between group social interactions (e.g. [45].

**Table 1:** Potential importance of six classes of factors on three scales of movement

	Step	Path	Life-history Phase
Sensory Perception	*** Sight, sound, smell: determines exact route taken	* Long distance perception	0
Movement Ability	*** Species specific locomotor abilities, also related to type of terrain moving through	** Endurance	* In conjunction with lifespan, places an upper limit on the area an individual can exploit
Ecological Niche	*** Food, shelter, predator avoidance	*** Food, shelter, predator avoidance	*** Food, shelter, predator avoidance
Social Interactions	*** Attraction & avoidance of immediate group members	*** Attraction and avoidance of non group members, territoriality	*** Attraction and avoidance of non group members, territoriality, seasonal breeding
Memory	* Direction of travel towards distant goal	*** Planned movement to known resources	* Seasonally changing resources

One to three *’s indicate the relative importance of the factors at each scale, and therefore our ability to draw inference about them based on observed patterns of movement

In this paper, we discuss how movement patterns fit within a multi-scale movement syndrome (MSMS) framework. This type of comparative approach to studying movement patterns is not only useful as a reflection of evolutionary adaptations, but also to understand their role in ecological processes (e.g. predators, seed dispersers) and spread of zoonotic diseases [14]. While there are dozens of metrics that might be used to describe animal movement, recent comparative work has shown the utility of combining relatively few complementary metrics to describe and contrast movement syndromes, where a group of movement patterns occur consistently together. For example, Sequeira et al. [44] found broad convergence across marine megafauna in the description of their movement steps with two metrics, while Abrahms et al. [1] found that a combination of five long-term (life-history phase scale) movement metrics revealed four distinct movement syndromes: migration, nomadism, territoriality, and central place foraging. Here we suggest that the movement syndrome approach of Abrahms et al. [1] can be applied at multiple scales. At the step level, we look at not only the size and angle of steps (e.g. [44], but also the shape of the distributions of these parameters. At the path level, we combine point clusters that reflect general movement phases [36] with two-dimensional measures (e.g., distance, straightness, sinuosity). At the life-history phase level we make our comparison between range-resident adults, but recognize the potential to do this for other parts of a species’ life history (e.g. dispersal, migration, natal range). At the largest scale, we suggest that the lifetime tracks of animals can be described by syndromes that relate to differing behavioral strategies.

As a test case for exploring multi-scale animal movement syndromes, we analyzed data from four sympatric frugivorous mammal species: kinkajous (*Potos flavus*), white-nosed coatis (*Nasua narica*), white-faced capuchins (*Cebus capucinus*), and black-handed spider monkeys (*Ateles geoffroyi)*. We hypothesized that the unique ecology and locomotor adaptations of these species (Table 2) would result in species-specific syndromes at all scales of movement. This comparative approach allows us to identify differences between the biology of the species and predict how it should affect their movement patterns. Kinkajous are strictly arboreal and have limited gap-crossing ability, therefore we expect them to follow more sinuous tracks and have the smallest turning angles because of limited route options between feeding sites. Because coatis rely primarily on olfaction to find food, we expect them to have larger turning angles, more sinuous paths, and shorter average step lengths than the visually oriented capuchins [23, 24]. The long arms of spider monkeys help them cross gaps easier than other species (i.e. through semi-brachiation), so they experience a more continuous canopy structure than quadrupedal capuchins or kinkajous. Therefore, we predict spider monkeys would have straighter, less sinuous travel paths with smaller turning angles at the step level. We also predicted that spider monkey step length distribution would be right skewed compared to other frugivores (i.e., more long steps) because of their highly efficient mode of locomotion. Finally, because capuchins and coatis consume more invertebrates, we expected that they would spend more time in area restricted search (ARS) than spider monkeys and kinkajous, which spend more time in fruiting trees, resulting in point clusters.

**Table 2:** Basic movement statistics and ecological characteristics of 46 animals from four frugivorous species studied. Weight is an average of adult animals caught during this study. N is number of individuals. Home range size and average distance moved per individual is plotted in Additional file 1: Fig. S1. Daily distance move is the average across all animal-days, individual averages are reported in Additional file 1: Table S3

Species	Sex	Mass (kg)	n	GPS fixes	Daily distance moved (m)	Home Range (ha)	Group Size	Activity Period	Substrates used	Locomotory adaptations
Kinkajou	M	2.96 ± 0.67	7	62,628	3736.7 ± 379.3	44.2 ± 17.9	1	Nocturnal	Arboreal	Climber, can’t jump across gaps
Kinkajou	F	2.70 ± 0.45	7^#^	83,093	2983.4 ± 277.9	18.4 ± 6.5	1	Nocturnal	Arboreal	Climber, can’t jump across gaps
White-nosed coati	M	6.11 ± 0.98	6	1,14,495	3678.6 ± 471.7	131.7 ± 87.1	1	Diurnal	1^o^Terrestrial	Mostly terrestrial, arboreal travel uncommon
									2^o^Arboreal	
White-nosed coati	F	4.69 ± 0.61	10	1,42,031	3874.2 ± 285.1	161.5 ± 85.4	6–35	Diurnal	1^o^Terrestrial	Mostly terrestrial, arboreal travel uncommon
									2^o^Arboreal	
White-faced capuchin	M	3.58 ± 0.93	4	62,860	4024.3 ± 117.8	132.4 ± 68.8	9–18	Diurnal	1^o^Arboreal	Climber, jumps across gaps, regularly walks on ground
									2^o^Terrestrial	
White-faced capuchin	F	2.74 ± 0.15	4	56,245	3465.1 ± 287.1	103.8 ± 29.1	9–18	Diurnal	1° Arboreal	Climber, jumps across gaps, regularly walks on ground
									2^o^Terrestrial	
Black-handed spider monkeys	M	8.25 ± 0.74	3	45,301	3301.6 ± 104.4	850.7 ± 135.3	1–37*	Diurnal	Arboreal	Brachiating climber, jumps across gaps
Black-handed spider monkeys	F	7.99 ± 1.16	5	1,04,949	2985.0 ± 410.0	713.6 ± 521.2	1–37*	Diurnal	Arboreal	Brachiating climber, jumps across gaps

#Two animals were repeated in the two field seasons

*Spider monkeys live in fission-fusion societies with flexible sub-grouping. The total population size on the island is estimated at 52, the range of observed group sizes during study period was 1-37

## Methods

### Study site

This research was conducted at the Smithsonian Tropical Research Institute on Barro Colorado Island (BCI), Panama (9°10′ N, 79°51′ W) from December 2015- February 2016 and December 2017- February 2018. BCI is a 1560 ha island of semi-deciduous tropical lowland forest that was isolated from the mainland in 1914 when the Chagres River was dammed to form Lake Gatun and the Panama Canal. The average annual rainfall at the site is 2600 mm/year, 90% of which falls between May and December [13]. Patterns of fruit availability at the site relate to this uneven distribution of rainfall: fruit availability is high during the dry season and into the early wet season, and is lowest during the late wet season [31, 50]. Half of BCI is covered by relatively young forest (at least 100 years old) that is still growing back from agricultural clearing. The remainder of the forest is older and is not thought to have undergone substantial anthropogenic disturbance in the last 200–400 years.

### Animal capture and collaring

Prior to the start of data collection, we captured, chemically immobilized, and fit all adult animals with GPS collars (see Additional file 1: Table S1 for details), each of which safely recovered from anesthesia. The Institutional Animal Care and Use Committees (IACUC) at the Smithsonian Tropical Research Institute (Protocol number 2014-1001-2017, 2017-0605-2020 and 2017-0912-2020) and the University of California, Davis (Protocol number 18239) approved all animal handling and collaring procedures.

### Animal tracking

Collars (e-Obs, https://e-obs.de/) were programmed to collect a burst of six consecutive (1 Hz) GPS locations every four minutes during the animal’s active periods: 23:00–6:30 for the nocturnal kinkajous, 06:00–18:30 for coatis, and 06:00–18:00 for capuchins and spider monkeys. 3D acceleration (ACC) was recorded at one-minute intervals for 5 s to determine activity profiles. From December 2015 to March 2016, collars on kinkajous and coatis were ACC informed [6], with the threshold set to 1000 mV. This allowed the collars to save battery life by attempting fewer GPS fixes when the animal was not moving, as judged by the ACC. All collars were programmed to timeout if they did not acquire a fix after 90 s. Details regarding the size and models of the collars are available in Additional file 1: Table S1.

The last fix of each burst consistently had the best horizontal accuracy measurement, therefore only the last fix of each burst was used for all analyses. Duplicates were removed and outliers were identified by altitude (i.e., height above ellipsoid) values less than or equal to 21 or greater than 244, suggesting impossible values and thus being marked as outliers. This corresponds to the first quartile minus twice the interquartile range and the third quartile plus twice the inter quartile range respectively. Subsequent outlier detection and removal was done using the ctmm package in R [8] using error information, straight line speeds, and distances from the median latitude and longitude to manually identify outliers via the outlie() function. Furthermore, obvious impossible locations, such as location estimates in the water were marked as outliers using a polygon of the boundary of the island.

The ACC informed collars were set to only take GPS points when an animal was moving (according to acceleration data). To reconstruct a continuous track that included these stationary positions we interpolated fixes at times when the animals were inactive (below their ACC thresholds). The error on the interpolated positions was modeled to replicate the observed GPS error of a stationary collar in a tree and was drawn from a negative binomial distribution with a mean of 5.46 m and a dispersion parameter of 2.4 m. One female kinkajou, Chloe, was removed from the path and life-history phase analyses due to an early collar failure and short tracking period (18 days) compared to the other animals.

### Descriptive statistics for movement

We calculated a series of statistics to describe the patterns of movement of our GPS tracked animals at the step, path, and life-history phase scales (Additional file 1: Table S2). Our goal was to describe the different aspects of the patterns of movement at each scale through a series of complementary metrics, following the approach of Abrahms et al. [1]. At the step scale, we fit distributions to the step lengths and turning angles to estimate the parameters of interest. For step lengths, we used the Gamma distribution (shape parameter k and a scale parameter θ) and for turning angles the von Mises distribution (distribution is clustered around the location (i.e., mean direction) μ, with a measure of concentration, κ). Distributions were fit using the *MASS* and *circmax* packages respectively [30, 43].

To describe the daily paths we chose five metrics to describe the pattern and extent of movement: distance, straightness, sinuosity and the proportion of time in clusters or area restricted search (ARS) (defined in Box 1, [2]. Daily distance traveled was calculated by summing the straight-line distances between consecutive fixes, and days where missed fixes resulted in less than hourly data were omitted. The straightness index and sinuosity index were calculated using the *trajr* packge in r [32], with the step length set to the mean value across all study animals of a species. We also used the characteristics of the track to categorize the behavior of animals based on movement pattern as: either clusters, ARS, or traveling. This characterization was primarily based on the first passage time statistic calculated in the R package *adehabitatLT* [9]. For a given 12 min window, we considered locations from animals that had moved less than 15 m a cluster, those that had moved between 15–30 m ARS, and those that moved > 30 m traveling. These criteria were based on our a-priori definition of a feeding bout as being at least 12 min and is designed to characterize locations where animals were likely within the same tree crown (< 15 m) for at least three fixes (12 min) as a cluster. During the course of this study, all three species intensively fed on *Dipteryx oleifera* trees, which are large-crowned trees that contain a large number of fruits. While animals feed in trees for shorter bouts, we considered these less important, and more similar to area restricted search. Because first passage time values could not be generated at the start or end of a day’s movement we also calculated two additional statistics (ETA and TAU) using the *smove* r package [19]. ETA and TAU are parameters of the best fit correlated velocity model, corresponding to the root mean squared velocity and the timescale of autocorrelation respectively. Their values show similar patterns to first passage time, but can be calculated on all time points. We then used a hierarchical clustering algorithm (JMP, SAS, Cary, North Carolina) to group the locations without first passage time values into the appropriate behavioral categories based on similarity of values for ETA and TAU. This algorithm models hierarchical relationships in the data by successively merging similar clusters together, which we did until all the data are in three clusters representing moving, ARS, or a point cluster.

We followed the protocol of Abrahms et al. [1] to calculate the following life-history phase level statistics: turn angle correlation, residence time, time-to-return, volume of intersection, and maximum net squared displacement (Box 1) using the *adehabitatLT* and *adehabitatHR* packages in R [9]. We calculated home range values using the *ctmm* package [8] which provides a model based approach for analyzing animal movement data as a continuous-time stochastic process. The best fit models were used to estimate biologically meaningful model parameters (position autocorrelation/home range crossing timescales and mean speed of the Gaussian process) and to generate autocorrelated kernel density estimates (AKDE) home range.

### Comparing individuals and species

For each scale of movement (step, path, and life-history phase) we used two approaches for comparing the descriptive statistics across species in the JMP software program (SAS, Cary, North Carolina). First, we used ANOVAs to quantify the proportion of variation in the metrics attributable to individuals vs. species. Second, we used Principal Components Analysis (PCA) to determine if individuals formed clusters that could be considered syndromes based on the similarity of their movement patterns. At the step level, we calculated the distribution parameters for turn angles and step length for each individual over the entire study and used these for both the ANOVA and PCA. At the path level we created PCA’s first using each individual animal-day as our sample unit and then again using average values for each individual animal across the entire study. In addition to the cross-species comparison, we also created PCA’s of the daily data for each species to search for individual level movement syndromes [22]. For the life-history level, we analyzed data for each individual to compare species. For these analyses the straightness measure was log transformed. For daily path statistics we considered only those paths with at least 100 locations.

Box 1. Glossary of terms used in the paper.

#### Step measurements

- *Step Length*: Distance that an animal moves between two consecutive GPS fixes. Characterized by a Gamma distribution parameterized by values for scale (*θ*) and shape (k).
- *Turn Angle*: Angle that animal turns as defined by 3 consecutive GPS fixes. Characterized by a von Mises distribution parameterized by values of concentration (*k*) and mean direction (μ).

#### Path measurements

- *Distance*: The distance moved per day as a sum of step lengths.
- *Straightness*: A measure of orientation efficiency calculated as the ratio between the distance from the starting point to the goal and the path length travelled to reach the goal (see Eq. 1 in [2]
- Sinuosity: The tortuosity of a search path, a function of both the mean cosine of turning angles and step length (see Eq. 8 in [2]
- *Cluster*: A location intended to represent a feeding or resting site, defined here as a movement of < 15 m in 12 min.
- *Area restricted search (ARS)*: A location intended to represent an animal searching for resources, defined here as a movement of > 15 but < 30 m in 12 min.
- *Travel*: A location intended to represent an animal moving relatively rapidly, defined here as a movement of > 30 m in 12 min

#### Life-history phase Measurements (from [1])

- *Turn angle correlation*: The sum of squares of distances between N successive turn angles.
- *Residence time*: Number of hours the animal spends inside a circle of a given radius centered on each location without leaving the radius for more than a specified cut-off time; we used a radius of mean step length and a 12 h cut-off time.
- *Time-to-return*: The number of hours the animal spends beyond a specified cut-off time before its return to a circle of a given radius centered on each location; we used a radius of mean step length and a 12 h cut-off time.
- *Volume of intersection*: The overlap between monthly 95% kernel density home ranges serving as a measure of home range stability.
- *Max net squared displacement*: Maximum squared Euclidean displacement from the first relocation of the trajectory over the full course of the trajectory; this value is scaled for each individual by dividing by the smallest MNSD observed for its species.

## Results

### Animal tracking and behaviors

We obtained 671,602 GPS locations for 46 animals from 4 species (Table 2). Below we describe the patterns found in these movements at three scales: step, daily path and life-history.

### Step-level patterns

**Fig. 2:**
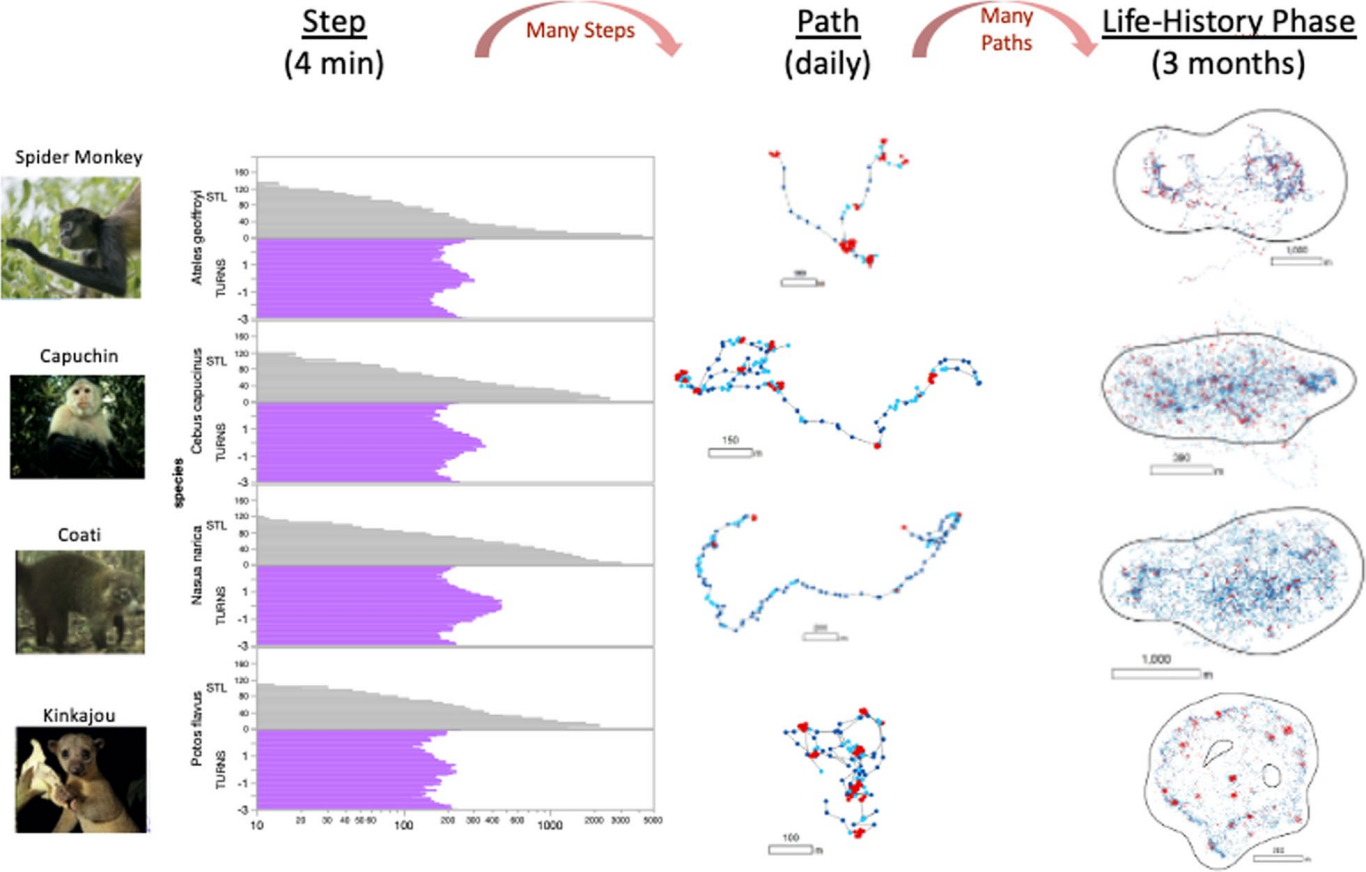
Example data for four species showing three scales of movement. The distribution of step-level measurements for turning angle (TURNS) and step length (STL) is shown for all animals combined. One example daily path is illustrated for one individual of each species showing behavioral phases segmented into traveling (dark blue), area restricted search (light blue) and clusters (red). Example life-history phase level data shows the 95% AKDE home range estimate and all recorded tracks for one individual. The daily paths exemplify the three primary path-level movement syndromes: spider monkey (high Cluster, high straightness, low distance), kinkajou (high sinuosity, low straightness, low distance) and coati-capuchin (high distance, higher ARS)

When considering the finest scale measures of animal movement, we found relatively few species-specific tendencies. We found moderate amounts of variation attributable to species for the scale of the step lengths (*θ*) and concentration of the turn angles (*k*), but little or none for the direction of the turn angles (μ) or the shape of the step length distribution (k) (Fig. 2, Additional file 1: Table S3). Pairwise comparisons showed capuchins to have significantly (17%) larger step lengths *θ* than the other three species (t test, t = 2.014, *p* < 0.05) and kinkajous to have a significantly (26%) smaller turn angle concentration (i.e., more short turns) than the other three species (t test, t = 2.015, *p* < 0.04).

**Fig. 3:**
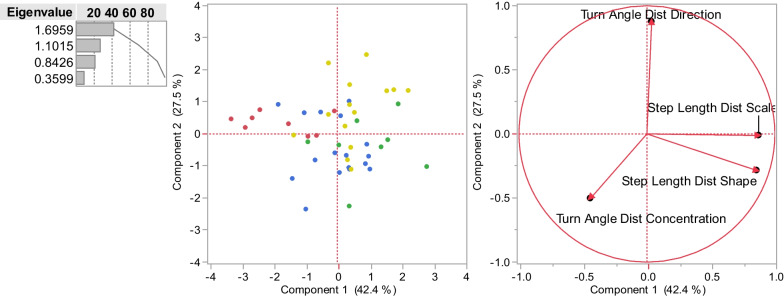
PCA of step level movement for 46 individuals of four species derived from four statistics (see Box 1) describing the distribution of turn angles and step lengths. There is high overlap across species and no obvious grouping into syndromes. Each colored dot represents an individual, with red for spider monkeys, green for capuchins, blue for coatis, and yellow for kinkajous

The four step-level metrics were described by two principal components with an eigenvalue > 1 which described 69.6% of the total variance (Fig. 3). The first component weighted both step length values positively and turn angle values negatively, reflecting the trend for faster movement to have shallower turns, while the second component reflected turn angle direction. However, this pattern was seen across all species, and individuals of different species were well mixed across both components, revealing no obvious species-level trends in step-scale movement.

### Daily movement patterns

We illustrated daily activities for each individual (example individuals in Fig. Additional file 1: S2A), by segmenting movements into three phase types (cluster, ARS, and traveling), as well as species level daily routines (Fig. Additional file 1: S1B) and total activity budgets (Fig. Additional file 1: S1C). Spider monkeys spent the most time in clusters, and the least time in ARS or traveling. Capuchins and coatis were similar in having relatively fewer clusters, more ARS, and more traveling. Kinkajous used clusters more than coatis and capuchins, but less than spider monkeys, and the three behaviors were relatively evenly spread across their nightly routine.

**Fig. 4:**
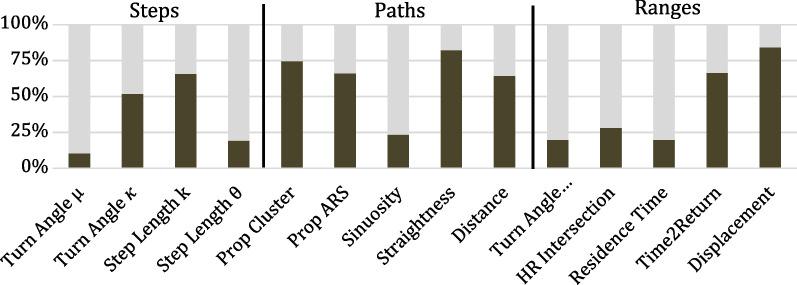
Percentage of variation explained by classification at the species (black) or individual (grey) level for 14 movement measures across three scales. All factors had had a significant (*p* < 0.05) amount of variation attributable to species-level differences except turn angle μ. Full ANOVA results are presented in Additional file 1: Table S2

**Fig. 5:**
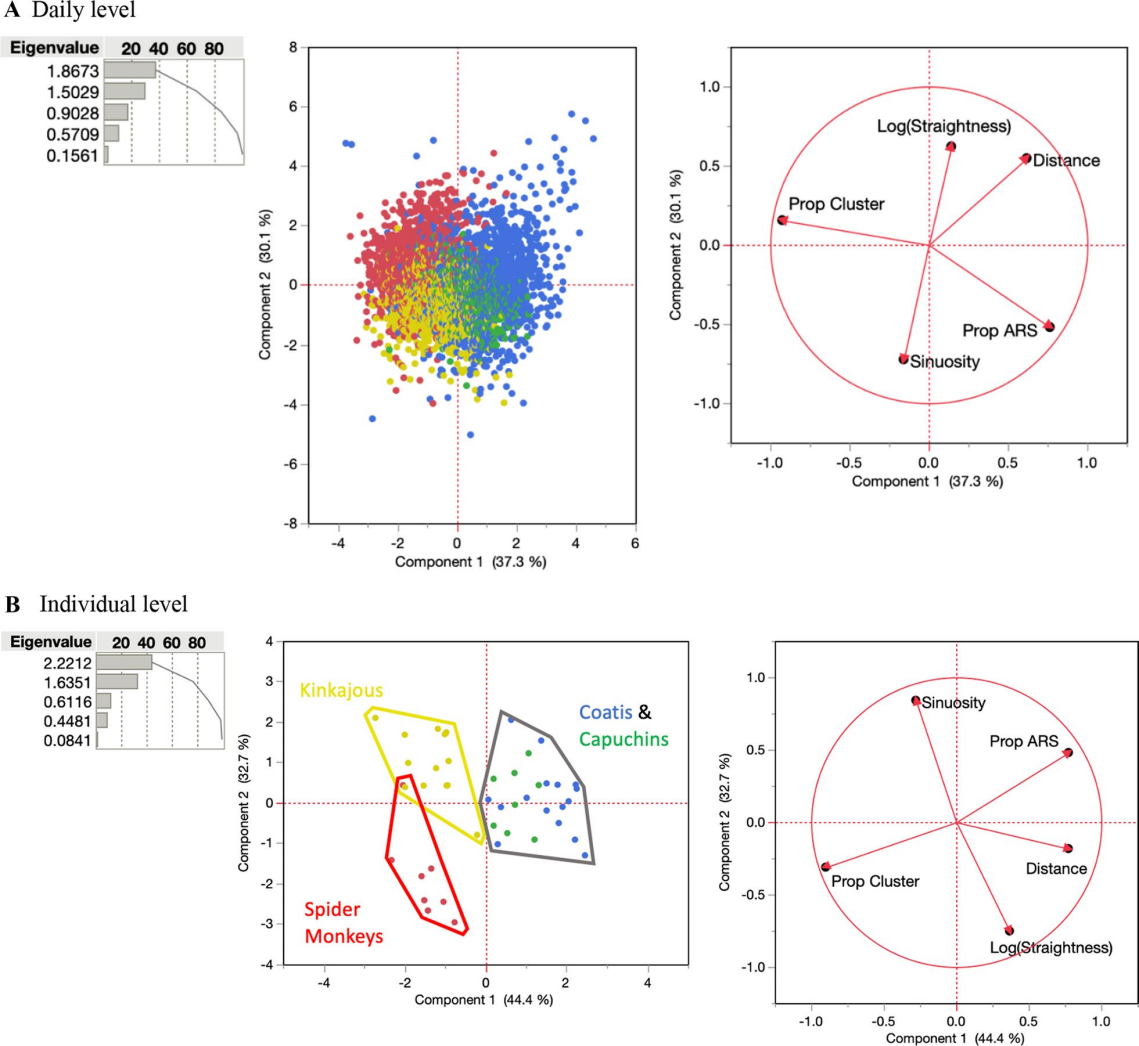
PCA of statistics (see Box 1) describing the daily movement paths for each day (**A**) and averaged per individual (**B**) with spider monkeys red, capuchins green, coatis blue, and kinkajous yellow. Colors represent species and are the same in each graph, as noted by cluster boundaries drawn in B

The daily distance moved was significantly different across species (means: kinkajou 3.3 km, coati 3.8 km, capuchin 3.7 km, and spider monkey 3.1 km, F_3,42_ = 7.39, *p* = 0.0007), although individual-level differences accounted for the majority (58%) of the variation (Additional file 1: Table S3). The five metrics (distance, sinuosity, straightness, cluster behavior, ARS behavior) we used to describe daily movement each had significant differences across species, with species-specific behaviors explaining 23–82% of the variation (Figs. 4, 5, Additional file 1: Table S3).

Species level PCA’s showed little evidence of individual-level syndromes (i.e., personalities, Additional file 1: Fig. S3). A PCA using these five metrics for all 3197 days of animal movement separately shows some species level trends, but substantial overlap (Fig. 5A). However, when taking average values for these movement metrics across each individual, species-level trends become more obvious, with separate clusters for spider monkeys, kinkajous, and one for coatis and capuchins together (Fig. 5B). Together, the two principal components with an eigenvalue > 1 described 77.1% of the variation. Based on the factors loading the two primary PCA axes we can characterize three syndromes (illustrated in Fig. 2). First, compared to other species, the kinkajou syndrome had higher sinuosity, lower straightness, shorter distance, higher cluster, and low ARS. Second, the spider monkey syndrome had lower sinuosity, lower straightness, shorter distance, higher cluster, and lower ARS. Finally, the coati-capuchin syndrome had longer distance and higher ARS.

### Life-history scale movement

We found very large variation across species in home range size (means: kinkajou 29 ha, coati 168 ha, capuchin 117 ha, spider monkey 774 ha (F_3,42_ = 29.8, *p* < 0.0001, Table 2, Additional file 1: Fig. S1, Table S2). Similarly, we found significant differences across species for all five life-history phase-level metrics identified by Abrahms et al. [1], although only two (time to return, max net squared displacement) had > 50% of variation attributable to species (Fig. 4, Additional file 1: Table S3).

**Fig. 6:**
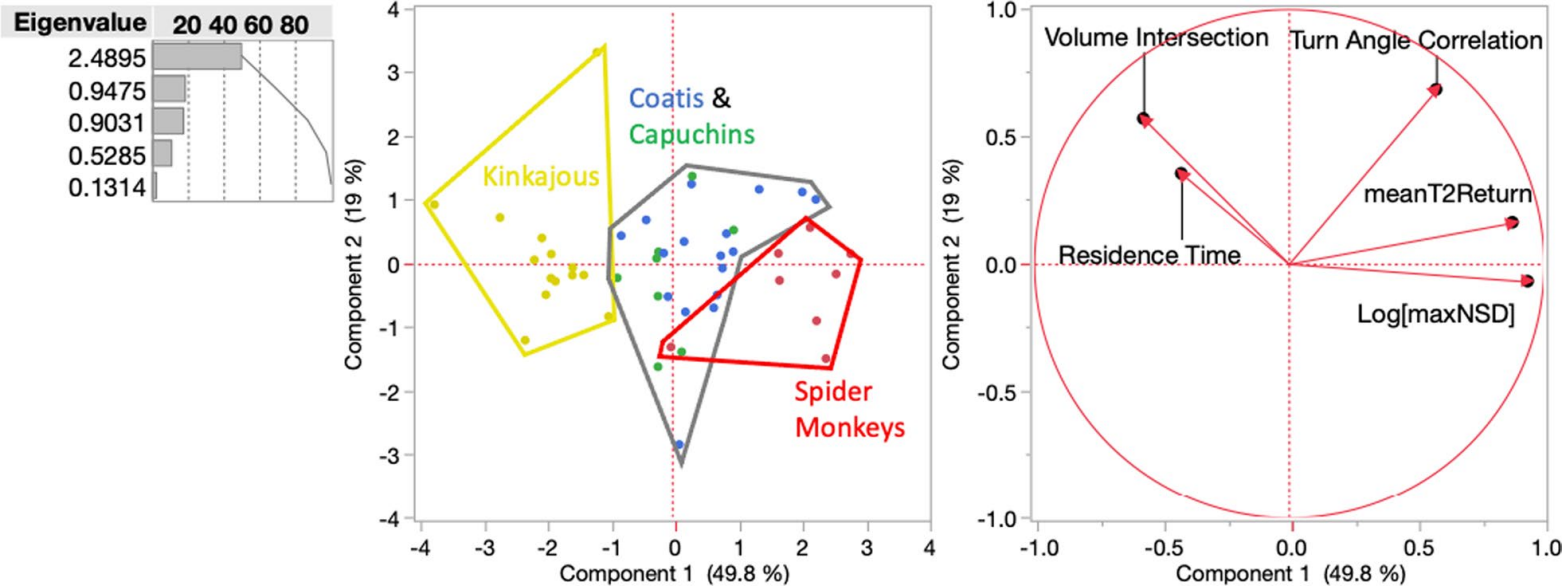
PCA of individual values for life-history phase level movement syndromes with spider monkeys red, capuchins green, coatis blue, and kinkajous yellow. The outlier animals were Ben Bob (kinkajou), Pliny (coati), and Emma (spider monkey)

A PCA analyses of these five metrics for all individuals revealed similar clusters to the daily syndromes (kinkajous, spider monkeys, coatis and capuchins) (Fig. 6). The first two principal components described 68.8% of the variation. This separation between species was primarily driven by the first principal component with spider monkeys having high values for net squared displacement and time to return, and low values for range volume intersection and residence time. Kinkajous had opposite tendencies, while coatis and capuchins fell in-between kinkajous and spider monkeys.

## Discussion

In this paper, we propose a multi-scale movement syndrome (MSMS) framework for comparing patterns of animal movement across species and apply it to a study of four sympatric frugivorous mammals. We found subtle differences between species at the step level, but three distinct movement syndromes at higher levels of spatial organization. Kinkajous and spider monkeys each assorted into their own species-specific movement syndrome, while coatis and capuchin monkeys clustered together into a shared movement syndrome at the larger scale levels of analysis. These results suggest that only some of the species-specific traits we expected to drive syndromes are important. Predictions made based on movement and sensory capabilities were not supported, while those based on feeding ecology were.

### Scales of movement patterns

We suggest that there is a natural four-level hierarchy that can be considered when comparing animal movements, with steps accumulating into daily paths, which form a life history phase (e.g., home range, dispersal, migration), that eventually become the lifetime track of an individual. This extends the framework proposed by Nathan et al. [37], which has steps forming phases (i.e. distinct behavior types), which then form lifetime tracks. The addition of daily (path) and monthly/yearly (life-history phase) levels to this framework are useful for describing meaningful scales of variation that match naturally occurring cycles, as well as traditionally used metrics of animal movement and space-use (i.e., home range). While the behavioral phases described by Nathan et al. [37] are important units of movement, the variation in their duration and the specificity of the behaviors they describe can make it difficult to compare them directly across species. We instead suggest that generic behavioral classifications (i.e., the clusters and ARS in our analysis) are used to describe differences in daily movement paths, rather than be the scales of movement themselves. Finally, lifetime tracks are the natural largest scale for individual based tracking. As tracking technology improves (e.g., smaller batteries, solar power, and ear tag attachments) there is greater potential to record lifetime tracks, and we imagine future studies will be able to compare metrics describing the dispersal, migration, and home range phases of an individual’s life.

Most comparative studies of animal movement have, to date, relied on coarse-scale relocation data (e.g., 24 h sampling rate) and focused on emergent patterns of space-use, rather than the underlying process of movement [1, 44, 47, 48]. As more fine-scale tracking data becomes available, an opportunity exists to compare across spatial scales, from step → path → life-history phase → life-track, and determine where differences in species’ movement patterns arise. This multi-level approach we suggest also provides important biological insights. For example, the step and path levels are the most appropriate scales to consider questions associated with animal locomotion and sensory ability, but they may also prove useful for addressing questions about ecological niche, social factors, and animal memory (Table 1). More specifically, describing the daily paths used by animals highlights the diversity of movement strategies an individual or species can use. In the case of our four frugivorous mammal species, individuals tended to move in one of three syndromes on average, although there was high overlap, showing that on any day, each individual had the ability to move in a pattern typical of another species.

### How many ways to be a frugivore?

Our high resolution (4 min) tracking data collected simultaneously on four sympatric frugivores provides an excellent opportunity to evaluate the similarities and differences in movement at multiple scales, across species and individuals. At the step level we found no obvious species level syndromes when considering all metrics together, and the subtle differences were opposite of what we predicted: capuchins (not spider monkeys) had the longest step lengths and kinkajous had the fewest (not most) sharp turns. This suggests that the unique sensory and locomotor adaptations of these species were not different enough to result in consistently large differences in these most basic components of movement: step lengths or turning angles. However, step-level metrics are the most likely to be affected by sampling rate GPS error, and potentially even vertical movement (which we didn’t study, but see [20]), which could have obscured species differences. Other studies comparing step metrics across species have found differences in step length associated with phylogeny, ecology, and human disturbance for terrestrial species, but fewer differences across marine taxa [44, 47, 48]. That we found relatively minor differences across species, may be because the time scale we used was shorter or because the movements of the species in our study are indeed similar in terms of step length. The latter is supported by the fact that they moved similar distances per day. However, as these steps were integrated into daily paths and monthly ranges, several other differences became evident.

Both paths and life-history phases grouped into three movement syndromes for the four species we studied. Our expectations based on feeding ecology were supported: kinkajous and spider monkeys spent less time in ARS than coatis and capuchins. Capuchins and coatis both eat a mixed diet of fruit and invertebrates [12, 18, 41], while kinkajous and spider monkeys are more dedicated fruit-eaters [28]. Invertebrate hunting should result in more ARS, while feeding at fruit trees results in more cluster behaviors. On the other hand, our hypotheses about movement differences expected based on other factors were not consistently supported. In accordance with previous studies [24], we predicted significant differences in the path level movement of coatis and capuchins based on differences in their primary locomotion (arboreal vs. terrestrial), sensory mode (vision vs. olfaction), and reliance on fallen fruit (in trees vs fallen) [21, 24]. However, in this study, capuchins and coatis grouped together into a single movement syndrome characterized by longer paths with similar levels of sinuosity and straightness. The daily movement of kinkajous and spider monkeys were similar to each other in having paths that were shorter with more time spent in clusters and less in ARS compared to the other two species but differentiated into separate syndromes based on straighter paths of spider monkeys and more sinuous paths of kinkajous. This matches our expectations based on movement ability, with the highly sinuous paths of kinkajous resulting from their limited gap-crossing ability, and the less sinuous spider monkey paths enabled by excellent gap-crossing abilities [11]. While averaged path methods show clear movement syndromes, our plot of daily values reveals great individual variation, showing that individuals of each species can occasionally move in patterns more characteristic of another. We found little evidence of consistent individual-level tendencies (i.e. personalities, [22] showing that, on a given day, any of these animals could travel a path with the characteristics of one of the other syndromes.

At the life-history phase level, the four species again clustered into three syndromes, although in this case it was primarily along principal component 1 which contrasted long values for time to return and high displacement (spider monkeys) vs. long residence times and volumes of intersection (kinkajous). Coatis and capuchins grouped together in between the other two species. This is probably driven by the fact that all species travelled similar distances per day but had very different home range sizes. The larger home ranges of the spider monkey resulted in them having a higher displacement, longer time to return, and lower home range intersection values. While these ecological correlations are interesting, we recognize the limitations of only comparing four species, and hope future work will extend this. Additionally, the spider monkeys on BCI represent one social group, so might not be representative of how the species moves when surrounded by other social groups [10].

The movement patterns of these four frugivores could have ecosystem-level implications given their roles as seed dispersers. Due to their size and relatively high abundance, the four species we tracked are some of the most important seed dispersers in their ecosystem, together responsible for ~ 20% of animal dispersed seeds in Panamanian rainforests [34]. Animal movement rates and patterns interact with gut retention times to determine how far a seed will be dispersed by a frugivore [4]. The similar step level movements, and roughly similar average daily movement distances of the four species (~ 3–4 km), lead us to believe that they should have similar potentials to act as long-distance dispersers. The longer distances and higher straightness for daily paths of coatis and capuchins should result in longer seed movements than in the other two species. Other measurements of movement patterns (i.e., ARS, sinuosity) could also affect the exact location of seed deposition, especially in association with habitat preferences of the species. Future comparisons of the movement and gut retention times of additional species have the potential to better quantify the relative importance of different species in dispersing seeds.

### Syndromes, resource selection functions, and simulations

We suggest that MSMS is a useful approach for describing the patterns of movement in comparative analyses, with the scales representing different temporal resolutions. This is complementary to established approaches for studying the habitat or environmental features selected by moving animals—i.e., Resource selection functions (RSF). Here we briefly review the already established hierarchical scales of RSF’s to show where the parallel MSMS fit. The basic approach of an RSF is to compare areas used by a species or individual with those potentially available to them, and these can be applied at four different spatial scales: within patch, within home range, population, and geographic range [26]. Here we focus on Johnson’s 3rd order of selection (the spatial scale within a home range, [26]) because it matches our description of movement patterns by individuals. At this spatial scale, the used vs. available comparison to derive an RSF can also be applied at three different time scales. First, at the finest temporal scale, Step Selection Functions compare each movement of an animal to the locations it could have chosen given its typical step-level movement patterns [46]. Second, the Path Selection Functions use simulations of daily movement paths to generate ‘available’ locations that are compared with the actual paths [52]. Finally, the traditional range-level RSF’s generate random points within a home range boundary to compare with locations the animal actually used [5].

These existing RSF approaches (step, path, home range) parallel our hierarchy for MSMS (Fig. 1) and are useful for deriving the animal-habitat interactions (i.e., habitat preferences) that are part of the process that led to the movement patterns described by syndromes. Other processes that interact with habitat preferences to determine the patterns of animal movement include the movement capabilities of a species, their social environment, and internal factors like memory and hunger [36]. We think that recognizing the parallels in temporal scale between RSF’s and movement syndromes offers exciting opportunities to connect movement processes and patterns in future research. Additionally, as more long-term tracks become available, we see exciting potential for comparing changes in both resource selection and movement patterns over the lifetime of an animal.

The most promising tools to link RSF processes to movement syndrome patterns are simulations (e.g., agent-based models), which allow researchers to program movement rules at fine scales and see what patterns emerge over time. These mechanistic models allow scientists to evaluate the effects of smaller scale decisions on large scale movement, and test for the importance of intrinsic factors that are difficult to directly measure, like memory or navigation ability [3, 16, 33]. Agent based models are most realistic when properly parameterized from real movement data [51] and we suggest that the results from MSMS and RSFs are ideal for this purpose, while keeping in mind the appropriate temporal scales.

## Conclusion

The proliferation of high-resolution tracking data is providing unprecedented detail on the behavior of animals that is useful not only for species specific discoveries, but also for broader comparative analyses that can reveal universal ecological patterns and evolutionary drivers. Here we suggest a MSMS framework for making these comparisons, expanding the step-level approach of Sequeira et al. [44], and create a new path-level approach which complements the existing life-history phase-level syndromes proposed by Abrahms et al. [1]. Our application of this framework to a comparison of four frugivorous mammals revealed three movement syndromes at the path and life-history phase level, which were explained more by feeding ecology than by differences in the locomotor or sensory adaptations of the species. We highlight existing RSF models that work at the same step-path-life-history phase hierarchy of scales as our MSMS and suggest these two approaches could be combined through simulation studies to gain more insight into the biological causes and ecological consequences of animal movement.

### Supplementary Information

**Additional file 1**: **Fig. S1**. Movement data for 46 animals shows similar average daily movement paths across species but dramatically different scales of home range size. Species averages are presented in Table 2. **Fig. S2** Daily records of animal behavior from segmented movement tracks for four species are registered from every GPS fix (4min intervals) as shown for in daily results for one example individual of each species (A). Averaging these values across individuals also shows general daily routines with values averaged per hour (B) and total average activity budgets per species (C). The color scale for behaviors in (B) applies to all graphs. **Fig. S3** To test for individual level syndromes (i.e. personalities) we constructed PCA of daily values for path level movement metrics for each species, color coded per individual. There was little separation of individuals. **Table S1** Capture details for the animals in the study. The collars for N. narica and A. geoffroyi included an electronic mechanism to automatically fall off after the study, while the collars for the other two species had weak points built in to ensure they would eventually break apart. Telazol consisted of 50 mg/ml tiletamine HCL and 50 mg/ml zolazepam HCL. **Table S2** ANOVA results on the movement stats at three scales for 48 individual animals across four species. **Table S3** Home range statistics from the Continuous Time Movement Model for 46 animals of 4 species. Data come from either the 2015–16 or 2017–18 field seasons, two animals were tracked in both seasons. All best fit movement models for all individuals were OU-F models, with a position and a velocity autocorrelation timescale. The Home Range Crossing timescale is the position autocorrelation timescale. Daily distance moved (meters/day) is the model estimated mean speed of the gaussian movement process

## Data Availability

The dataset supporting the conclusions of this article is available in the Movebank Data Repository DOI:10.5441/001/1.295 [29].
