# Supplementary material for: Multi-scale movement syndromes for comparative analyses of animal movement patterns
Source: Mov Ecol. 2023 Oct 4;11:61. doi: 10.1186/s40462-022-00365-y (PMC10552421; doi:10.1186/s40462-022-00365-y)
Supplement: Supplementary file 1 — Additional file 1: Fig. S1. Movement data for 46 animals shows similar average daily movement paths across species but dramatically different scales of home range size. Species averages are presented in Table 2. Fig. S2 Daily records of animal behavior from segmented movement tracks for four species are registered from every GPS fix (4min intervals) as shown for in daily results for one example individual of each species (A). Averaging these values across individuals also shows general daily routines with values averaged per hour (B) and total average activity budgets per species (C). The color scale for behaviors in (B) applies to all graphs. Fig. S3 To test for individual level syndromes (i.e. personalities) we constructed PCA of daily values for path level movement metrics for each species, color coded per individual. There was little separation of individuals. Table S1 Capture details for the animals in the study. The collars for N. narica and A. geoffroyi included an electronic mechanism to automatically fall off after the study, while the collars for the other two species had weak points built in to ensure they would eventually break apart. Telazol consisted of 50 mg/ml tiletamine HCL and 50 mg/ml zolazepam HCL. Table S2 ANOVA results on the movement stats at three scales for 48 individual animals across four species. Table S3 Home range statistics from the Continuous Time Movement Model for 46 animals of 4 species. Data come from either the 2015–16 or 2017–18 field seasons, two animals were tracked in both seasons. All best fit movement models for all individuals were OU-F models, with a position and a velocity autocorrelation timescale. The Home Range Crossing timescale is the position autocorrelation timescale. Daily distance moved (meters/day) is the model estimated mean speed of the gaussian movement process [file 40462_2022_365_MOESM1_ESM.docx]

Fig. S1. Movement data for 46 animals shows similar average daily movement paths across species but dramatically different scales of home range size. Species averages are presented in Table 2.

Fig. S2. Daily records of animal behavior from segmented movement tracks for four species are registered from every GPS fix (4min intervals) as shown for in daily results for one example individual of each species (A). Averaging these values across individuals also shows general daily routines with values averaged per hour (B) and total average activity budgets per species (C). The color scale for behaviors in (B) applies to all graphs.

A

B

C


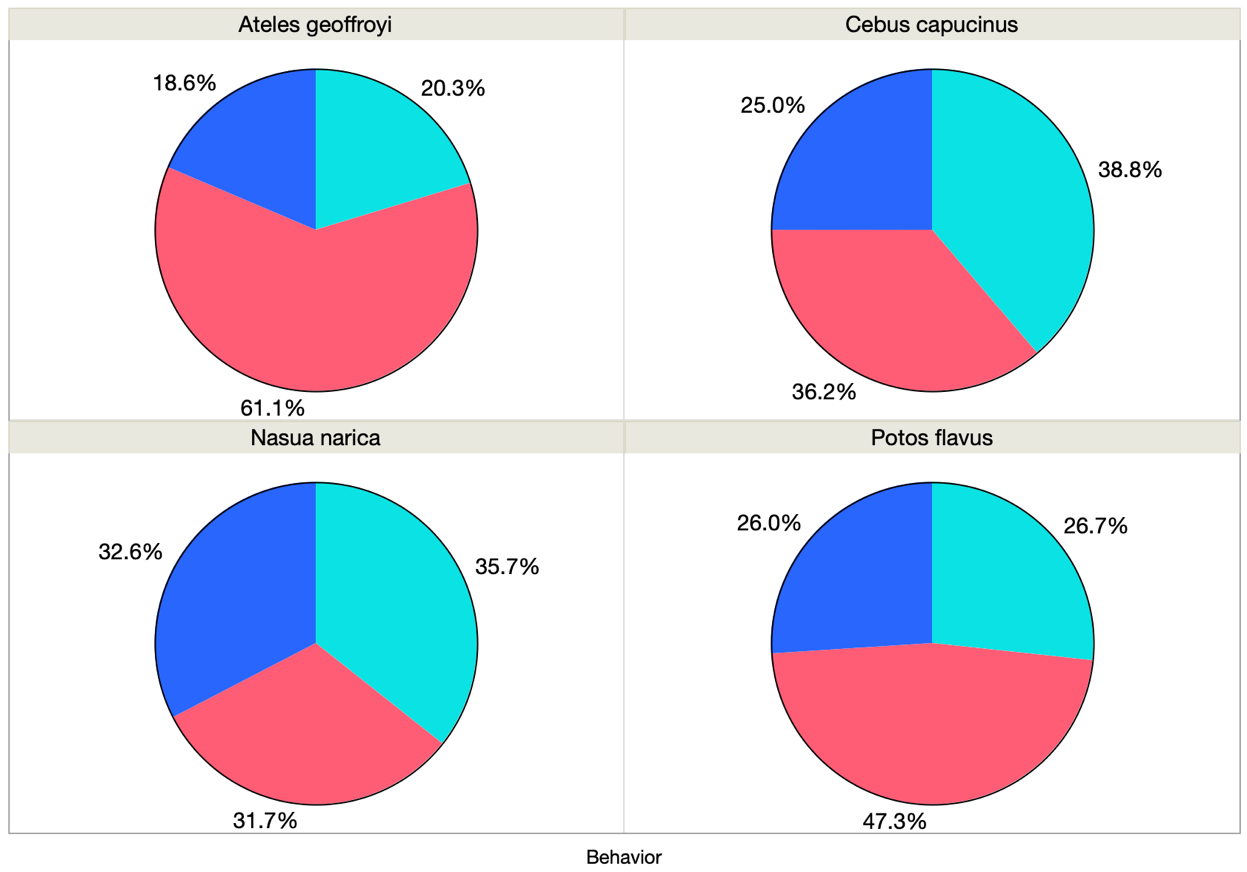


Fig. S3. To test for individual level syndromes (i.e. personalities) we constructed PCA of daily values for path level movement metrics for each species, color coded per individual. There was little separation of individuals.

1. Spider Monkeys

1. Capuchins

1. Coatis

1. Kinkajous

Table S1. Capture details for the animals in the study. The collars for N. narica and A. geoffroyi included an electronic mechanism to automatically fall off after the study, while the collars for the other two species had weak points built in to ensure they would eventually break apart. Telazol consisted of 50 mg/ml tiletamine HCL and 50 mg/ml zolazepam HCL.

| Species | Weight (kg  ±sd) | Capture Method | Drug | Dosage | eObs collar model | Collar Weight (g) | Collar % Body Weight |
| --- | --- | --- | --- | --- | --- | --- | --- |
| *N. narica* | M: 5.6±0.88, n=10  F: 4.7±0.63 n=10 | Terrestrial cage trap | Telazol® | 5 mg/kg | 1-C heavy | 206 | M: 3.7 F: 4.4 |
| *P. flavus* | M: 3.0 ±0.55, n=8 F: 2.7 ±0.36, n=9 | Arboreal cage trap | Ketamine & Xylazine | Ket:10mg/kg Xyl:5 mg/kg | 1-A | 81 | M: 2.7 F: 3.0 |
| *C. capucinus* | M: 3.6 ±0.93, n=4 F: 2.7 ±0.15, n=4 | Darting | Telazol® | 15 mg/kg | 1-A | 67 | M: 1.9 F: 2.4 |
| *A. geoffroyi* | M: 7.9 ± 0.9, n=4 F: 8.2 ± 1.17, n=4 | Darting | Telazol® | 15 mg/kg | 1-C light | 166 | M: 2.1 F: 2.0 |

Table S2. ANOVA results on the movement stats at three scales for 48 individual animals across four species.

|  | **DF** | **Sum of Squares** | **Mean Square** | **F Ratio** | **Prob > F** | R squared |
| --- | --- | --- | --- | --- | --- | --- |
| **Step Level** |  |  |  |  |  |  |
| **Turn Angle μ** |  |  |  |  |  |  |
| Species | 3 | 0.570 | 0.190 | 1.706 | 0.180 | 0.104 |
| Individual | 45 | 4.900 | 0.111 |  |  |  |
|  |  |  |  |  |  |  |
| **turn angle 𝜅** |  |  |  |  |  |  |
| Species | 3 | 0.080 | 0.027 | 15.755 | <.0001* | 0.518 |
| Individual | 45 | 0.075 | 0.002 |  |  |  |
|  |  |  |  |  |  |  |
| **Step Length k** |  |  |  |  |  |  |
| Species | 3 | 1.295 | 0.432 | 28.162 | <.0001* | 0.658 |
| Individual | 45 | 0.674 | 0.015 |  |  |  |
|  |  |  |  |  |  |  |
| **Step Length θ** |  |  |  |  |  |  |
| Species | 3 | 0.001 | 0.000 | 3.526 | 0.0225* | 0.194 |
| Error | 45 | 0.004 | 0.000 |  |  |  |
|  |  |  |  |  |  |  |
| **Path Level** |  |  |  |  |  |  |
| **prop cluster** |  |  |  |  |  |  |
| Species | 3 | 0.49 | 0.16 | 41.24 | <.0001* | 0.762 |
| Individual | 45 | 0.17 | 0.00 |  |  |  |
|  |  |  |  |  |  |  |
| **prop ARS** |  |  |  |  |  |  |
| Species | 3 | 0.19 | 0.06 | 27.32 | <.0001* | 0.691 |
| Individual | 45 | 0.10 | 0.00 |  |  |  |
|  |  |  |  |  |  |  |
| **sinuosity** |  |  |  |  |  |  |
| Species | 3 | 0.00 | 0.00 | 4.29 | 0.0100* | 0.520 |
| Individual | 45 | 0.02 | 0.00 |  |  |  |
|  |  |  |  |  |  |  |
| **Straightness (log transformed)** |  |  |  |  |  |  |
| Species | 3 | 19.23 | 6.41 | 64.94 | <.0001* | 0.727 |
| Individual | 45 | 4.15 | 0.10 |  |  |  |
|  |  |  |  |  |  |  |
| **Distance** |  |  |  |  |  |  |
| Species | 3 | 109146269.00 | 36382090.00 | 25.37 | <.0001* | 0.580 |
| Individual | 45 | 60237691.00 | 1434230.70 |  |  |  |
|  |  |  |  |  |  |  |
| **Range Level** |  |  |  |  |  |  |
| **Turn Angle Correlation** |  |  |  |  |  |  |
| Species | 3 | 0.003 | 0.001 | 3.601 | 0.0206* | 0.197 |
| Individual | 45 | 0.012 | 0.000 |  |  |  |
|  |  |  |  |  |  |  |
| **HR Intersection** |  |  |  |  |  |  |
| Species | 3 | 0.240 | 0.080 | 5.833 | 0.0019* | 0.285 |
| Individual | 45 | 0.610 | 0.014 |  |  |  |
|  |  |  |  |  |  |  |
| **Residence Time** |  |  |  |  |  |  |
| Species | 3 | 11969 | 3990 | 3.639 | 0.0198* | 0.236 |
| Individual | 45 | 48235 | 1096 |  |  |  |
|  |  |  |  |  |  |  |
| **Time2Return** |  |  |  |  |  |  |
| Species | 3 | 99324 | 33108 | 28.980 | <.0001* | 0.664 |
| Individual | 45 | 50268 | 1143 |  |  |  |
|  |  |  |  |  |  |  |
| **Displacement (log transformed)** |  |  |  |  |  |  |
| Species | 3 | 69.10 | 23.03 | 78.955 | <.0001* | 0.843 |
| Individual | 45 | 12.84 | 0.29 |  |  |  |

Table S3. Home range statistics from the Continuous Time Movement Model for 46 animals of 4 species. Data come from either the 2015-16 or 2017-18 field seasons, two animals were tracked in both seasons. All best fit movement models for all individuals were OU-F models, with a position and a velocity autocorrelation timescale. The Home Range Crossing timescale is the position autocorrelation timescale. Daily distance moved (meters/day) is the model estimated mean speed of the gaussian movement process.

| **Species** | **Name** | **Sex** | **Field Season** | **mass (kg)** | **Daily Distance Moved (m)** | **95% AKDE Home Range (ha)** | **Home Range Xing Time (hrs)** | **Start Date** | **End Date** | **N days** |
| --- | --- | --- | --- | --- | --- | --- | --- | --- | --- | --- |
| *P. flavus* | Abby | F | 2018 | 3.05 | 2980.6 | 23.15 | 2.50 | 12/15/15 | 4/1/16 | 108 |
| *P. flavus* | Abby | F | 2016 | 3.05 | 2953.6 | 29.30 | 2.67 | 12/15/17 | 2/22/18 | 69 |
| *N. narica* | Avery | F | 2018 | 4.8 | 3991.0 | 59.26 | 3.22 | 12/15/17 | 3/27/18 | 103 |
| *P. flavus* | Ben Bob | M | 2016 | 3.6 | 4317.6 | 80.33 | 2.90 | 12/15/15 | 3/4/16 | 80 |
| *C. capucinus* | Bob | M | 2018 | 2.5 | 4084.7 | 83.67 | 6.77 | 12/15/17 | 3/1/18 | 77 |
| *P. flavus* | Bonnie | F | 2016 | 3.3 | 3059.7 | 21.19 | 2.55 | 12/15/15 | 4/18/16 | 107 |
| *N. narica* | Carlsberg | M | 2018 | 4.5 | 3896.5 | 80.73 | 3.54 | 12/15/17 | 3/23/18 | 99 |
| *A. geoffroyi* | Chibi | M | 2016 | 7.75 | 3187.0 | 699.43 | 44.93 | 12/11/15 | 2/20/16 | 72 |
| *P. flavus* | Chloe | F | 2016 | 2.9 | NA | NA | NA | 12/15/15 | 1/3/16 | 19 |
| *N. narica* | Clementina | F | 2016 | 4.05 | 4052.6 | 323.03 | 15.80 | 12/14/15 | 3/18/16 | 96 |
| *C. capucinus* | Da Vinci | M | 2018 | 3.1 | 4149.5 | 228.25 | 16.13 | 12/15/17 | 3/30/18 | 106 |
| *P. flavus* | Eli | M | 2018 | 2.5 | 3659.2 | 36.99 | 2.55 | 12/15/17 | 3/4/18 | 78 |
| *N. narica* | Ellie | F | 2016 | 4.8 | 3659.9 | 85.71 | 5.59 | 12/14/15 | 3/3/16 | 81 |
| *A. geoffroyi* | Emma | F | 2018 | 6.8 | 2947.5 | 143.71 | 17.23 | 12/15/17 | 4/23/18 | 130 |
| *N. narica* | Fonta Flora | F | 2018 | 5 | 4555.8 | 187.61 | 11.87 | 12/15/17 | 3/26/18 | 102 |
| *N. narica* | Galena | F | 2018 | 4.3 | 3833.5 | 150.12 | 6.51 | 12/15/17 | 3/21/18 | 97 |
| *P. flavus* | Gamer | M | 2018 | NA | 3910.5 | 35.38 | 1.81 | 12/15/17 | 2/15/18 | 63 |
| *N. narica* | Gillian | F | 2016 | 5.05 | 3581.9 | 97.16 | 8.94 | 12/14/15 | 3/2/16 | 80 |
| *N. narica* | Goliath | M | 2018 | 7.2 | 4216.4 | 296.47 | 13.12 | 12/16/17 | 3/14/18 | 89 |
| *A. geoffroyi* | Greg | M | 2016 | 7.9 | 3391.4 | 960.21 | 65.15 | 12/11/15 | 2/20/16 | 72 |
| *C. capucinus* | Ibeth | F | 2016 | 2.7 | 3736.9 | 87.83 | 7.05 | 12/25/15 | 3/31/16 | 98 |
| *A. geoffroyi* | Inez | F | 2018 | 8.8 | 2724.1 | 1001.75 | 116.09 | 12/11/17 | 5/19/18 | 141 |
| *P. flavus* | Jeff | M | 2018 | 3.7 | 3159.7 | 33.74 | 2.81 | 12/15/17 | 2/22/18 | 64 |
| *P. flavus* | Judy | F | 2016 | 2.6 | 2835.9 | 14.70 | 2.63 | 1/28/16 | 3/12/16 | 45 |
| *A. geoffroyi* | Kyle | M | 2016 | 9.1 | 3326.4 | 892.74 | 53.40 | 12/11/15 | 3/28/16 | 109 |
| *A. geoffroyi* | Limon | F | 2018 | 9.5 | 2499.7 | 197.83 | 28.88 | 12/1/17 | 6/14/18 | 183 |
| *P. flavus* | Mario | M | 2018 | 2.2 | 3624.1 | 41.54 | 3.77 | 12/15/17 | 2/25/18 | 70 |
| *C. capucinus* | Martinelli | M | 2018 | 4.3 | 3881.1 | 81.10 | 6.48 | 12/15/17 | 3/12/18 | 88 |
| *C. capucinus* | Mimi | F | 2016 | 2.7 | 3687.7 | 147.54 | 13.99 | 12/15/15 | 3/12/16 | 89 |
| *P. flavus* | Molly | F | 2018 | 3 | 3587.8 | 19.88 | 1.21 | 12/15/17 | 2/14/18 | 59 |
| *C. capucinus* | Norah | F | 2018 | 2.6 | 3191.0 | 88.58 | 8.67 | 12/15/17 | 2/14/18 | 63 |
| *C. capucinus* | Olga | F | 2016 | 2.95 | 3244.6 | 91.25 | 9.09 | 12/15/15 | 2/20/16 | 67 |
| *N. narica* | Ornette | M | 2016 | 5.65 | 3126.0 | 99.41 | 6.50 | 12/14/15 | 2/20/16 | 69 |
| *N. narica* | Peter Nelson | M | 2018 | 7 | 4056.1 | 159.96 | 9.27 | 12/15/17 | 3/19/18 | 94 |
| *N. narica* | Pliny | M | 2016 | 6.1 | 3106.0 | 58.87 | 3.49 | 12/14/15 | 4/21/16 | 114 |
| *P. flavus* | Ripley | F | 2016 | 2.4 | 2713.9 | 9.47 | 1.10 | 12/15/15 | 4/7/16 | 112 |
| *P. flavus* | Ripley | F | 2018 | 2.4 | 2713.7 | 10.97 | 1.66 | 12/15/17 | 2/22/18 | 69 |
| *N. narica* | Riwaka | F | 2018 | 3.95 | 3777.7 | 99.49 | 7.02 | 12/15/17 | 3/28/18 | 95 |
| *N. narica* | Sahti | F | 2018 | 4.5 | 3887.0 | 148.17 | 7.34 | 12/15/17 | 3/21/18 | 94 |
| *N. narica* | Sofie | F | 2016 | 4.75 | 3602.8 | 184.65 | 10.66 | 12/14/15 | 3/10/16 | 88 |
| *N. narica* | Thelonious | M | 2018 | 6.2 | 3670.8 | 94.69 | 5.40 | 12/15/17 | 2/11/18 | 59 |
| *P. flavus* | Tony Stark | M | 2018 | 3.6 | 3749.3 | 37.46 | 3.35 | 12/15/17 | 2/19/18 | 66 |
| *C. capucinus* | Valoy | M | 2018 | 4.4 | 3982.0 | 136.69 | 9.87 | 12/15/17 | 3/12/18 | 88 |
| *A. geoffroyi* | Veruca | F | 2016 | 7.85 | 3203.6 | 895.43 | 75.09 | 12/11/15 | 4/2/16 | 114 |
| *N. narica* | Vielle | F | 2016 | 5.7 | 3800.2 | 279.49 | 14.30 | 1/13/16 | 4/6/16 | 85 |
| *A. geoffroyi* | Zola | F | 2018 | 7 | 3550.3 | 1329.67 | 68.32 | 12/13/17 | 6/1/18 | 163 |
